# Comment on Comparison of Powder Dustiness Methods

**DOI:** 10.1093/annhyg/met086

**Published:** 2014-01-29

**Authors:** Douglas E. Evans, Leonid A. Turkevich, Cynthia T. Roettgers, Gregory J. Deye

Editorial Note. Letters to the Editor are peer reviewed to ensure that the arguments are reasonable and clearly expressed. However, letters may express a particular opinion rather than a balanced interpretation. Authors of papers commented on are invited to reply, but neither the journal nor peer reviewers should be assumed to support the arguments made.

We have read with interest the recent work by the University of Wuppertal group (Bach *et al.*, 2013) on dustiness determination using the University of North Carolina (UNC) Dustiness Testing Device (Boundy *et al.*, 2006). We have referred to the UNC device as the ‘Venturi’ device (Evans *et al.*, 2013), as that describes the underlying dispersal mechanism; we continue with this terminology. The Wuppertal paper is presented in two parts. In Part 1, the dustiness of nine industrial powders was measured with the Venturi device, and results compared with their earlier measurements (Bach and Schmidt, 2008) using macroscopic techniques: EN 1505 standardized continuous drop (CEN 2006, 2013) and the commercial Heubach rotating drum and commercial Palas single drop. In Part 2, dustiness values for 11 pharmaceutical powders were determined solely with the Venturi device. We would like to comment on these Wuppertal results, especially in light of our previous and extensive use of the Venturi device for fine and nanoscale powders (Evans *et al.*, 2013).

Unfortunately, insufficient detail is provided on the provenance of the Wuppertal powders (Bach and Schmidt, 2008; Bach *et al.*, 2013), to allow an inter-laboratory comparison with identical materials. (By contrast, our measurements (Evans *et al.*, 2013) for Holland lactose of D_tot_ = 5.2 (0.4)% and D_resp_ = 0.9 (0.1)% are fully consistent with those of the UNC group (Boundy *et al.*, 2006), with D_tot_ = 5.1 (0.9)% and D_resp_ = 1.3 (0.5)% for the same material.) In the technique comparison, Part 1, of the Wuppertal study, only three Venturi measurements were made for each powder, and no ranges or statistics were reported. In the pharmaceutical, Part 2, of their study, five Venturi measurements were made for each powder, and standard deviations were reported, permitting some analysis of possible error. Finally, we observed an empirical correlation between respirable and total dustiness, as measured with the Venturi device, to hold for a wide range of powders (Evans *et al.*, 2013). It is informative to test that empirical correlation with these additional Wuppertal results.

With the Venturi device, we measured total and respirable dustiness for 27 materials (Evans *et al.*, 2013), primarily focusing on fine and nanoscale powders, but also included are several materials with micrometer-sized primary particles, the presumed size of the Wuppertal Industrial powders; their Al_2_O_3_ can be traced to Aloxite F-1200, with mean volume diameter d ~ 3.6 μm (Mark *et al.*, 1985). The relative standard deviation (RSD), D_tot_/D_tot_, of the total dustiness is plotted (Fig. 1) as a function of the measurement value, D_tot_. The National Institute for Occupational Safety & Health (NIOSH) RSDs (typically, *n* ≥ 6, with minor exceptions, see Evans *et al.*, 2013) are all small, except for one extremely low dusty material (Kemira TiO_2_); the RSDs (*n* = 9) for the five materials tested in the original UNC study (Boundy *et al.*, 2006) are similarly small. By contrast, the RSDs (*n* = 5) for the Wuppertal pharmaceutical measurements (Wupp-Pharm) are systematically higher and become increasingly poor at lower dustiness values.

**1:**
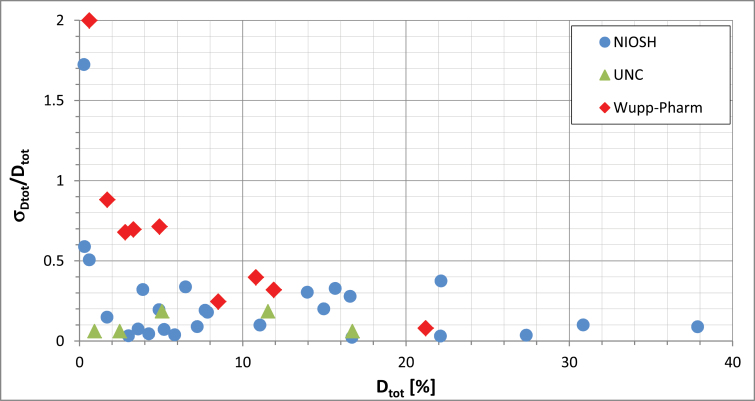
Relative standard deviation, D_tot_/D_tot_, of total dustiness as a function of total dustiness, D_tot_. NIOSH (Evans *et al.*, 2013), UNC (Boundy *et al.*, 2006), Wupp-Pharm (Bach *et al.*, 2013). All data derived from the Venturi device.

Similar higher RSDs obtain for the Wuppertal measurements of the respirable fraction (Fig. 2) and these, again, become increasingly poor at the lower dustiness values.

**2:**
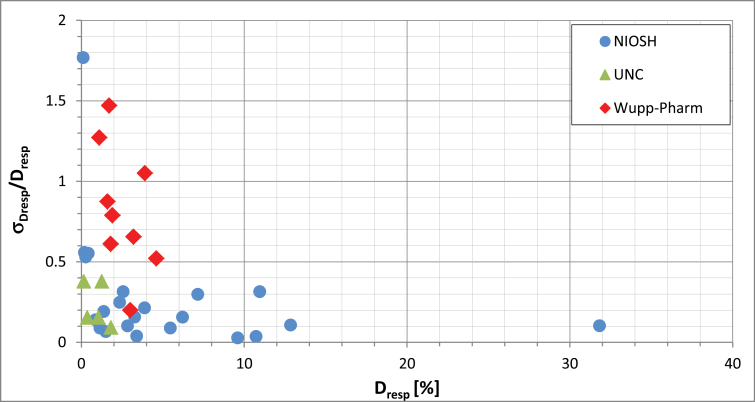
Relative standard deviation, D_resp_/D_resp,_ of respirable dustiness as a function of respirable dustiness, D_resp_. NIOSH (Evans *et al.*, 2013), UNC (Boundy *et al.*, 2006), Wupp-Pharm (Bach *et al.*, 2013). All data derived from the Venturi device.

We have found (Evans *et al.*, 2013) that particular care must be taken when gravimetrically measuring the less dusty powders; indeed, the least dusty powders are the most problematic for the Wuppertal group. We believe that this is the source of the ‘negative’ total dustiness that they report for Al(OH)_3_, and also for their having obtained physically unreasonable higher respirable than total dustiness values for erythromycin, metronidazole and tetracycline hydrochloride. We disagree with their statement ‘that the device is error-prone’; we and the UNC group have demonstrated that, with sufficient care, reproducible, physically reasonable results are obtainable with the device for a wide variety of powders. The UNC group also report impressive inter-instrument consistency with the device (Boundy *et al.*, 2006).

In our earlier work (Evans *et al.*, 2013), we found an unexpected, linear correlation between the respirable and total dustiness, as measured with the Venturi device. We have plotted (Fig. 3) the Wuppertal data, both for their industrial (‘Wupp-Ind’) and pharmaceutical (‘Wupp-Pharm’) powders, together with our earlier fine and nanoscale powders (‘NIOSH’) and the original UNC data (‘UNC’). The linear correlation is obeyed, with the exception of the very low dustiness Wuppertal materials: Al(OH)_3_, BaSO_4_-code B, metronidazole, erythromycin, lidocaine hydrochloride, and tetracycline hydrochloride.

**3:**
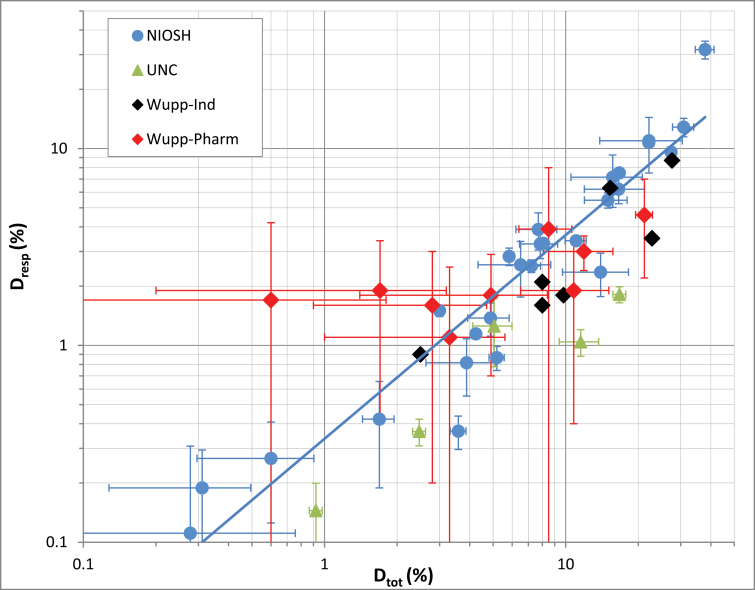
Scaling of respirable dustiness, D_resp_, versus total dustiness, D_tot._ Linear best fit to the earlier NIOSH fine and nanoscale powder data (Evans *et al.*, 2013). No error bars are plotted for the Wuppertal industrial powder data (Wupp-Ind) due to a lack of reported statistics. UNC (Boundy *et al.*, 2006), Wupp-Ind and Wupp-Pharm (Bach *et al.*, 2013). All data derived from the Venturi device.

Finally, we commend the attempt to correlate these four different dustiness methods. As we previously discussed (Evans *et al.*, 2013), the Venturi device disperses the powders at significantly higher Reynolds numbers than do the gravity-driven rotating drum or falling powder techniques and has unique value in simulating high energy dust dispersion operations in the workplace. Using the Wuppertal Venturi data (Bach *et al.*, 2013) and earlier data with gentler gravity driven techniques (Bach and Schmidt, 2008), we construct the following correlation tables (*r* = √*R* ^2^, simple linear regressions are calculated for *n* = 9 materials, using MS Excel) for the paired dustiness techniques.

Tables 1 and 2 do not suggest a consistent correlation between ‘any’ paired dustiness techniques, nor do they point to any one method as being anomalous. That the total (inhalable) and respirable correlations do not even mimic each other, motivates further research into the mechanics of powder dispersal and resulting dustiness. Since the Venturi technique disperses powders at high Reynolds number (Re ~ 10^4^, Evans *et al.*, 2013), whereas other techniques disperse at low Reynolds number (Re ~ 10^2^), there may be an intrinsic difference in dustiness due to the Reynolds number of dispersion. This is the subject of current investigation at NIOSH. We note that weak correlations were obtained between the EN 15051 rotating drum and EN 15051 continuous drop measurements, both gravity driven techniques at low Reynolds numbers, for nine industrial minerals (Pensis *et al.*, 2010).

**Table 1.** Correlation coefficients for total (inhalable) dustiness, measured by four dustiness techniques for nine industrial powders (Venturi measured in Bach *et al.*, 2013 and Heubach rotating drum, EN 15051 continuous drop and Palas single drop all measured in Bach *et al.*, 2008).

Device	Venturi	Heubach rotating drum	EN 15051 continuous drop	Palas single drop
Venturi	1.00	0.84	0.11	0.77
Heubach rotating drum		1.00	0.34	0.79
EN 15051 continuous drop			1.00	0.61
Palas single drop				1.00

**Table 2.** Correlation coefficients for respirable dustiness, measured by four dustiness techniques for nine industrial powders (UNC Venturi measured in Bach *et al.*, 2013 and Heubach rotating drum, EN 15051 continuous drop and Palas single drop all measured in Bach *et al.*, 2008).

Device	Venturi	Heubach rotating drum	EN 15051 continuous drop	Palas single drop
Venturi	1.00	0.72	0.80	0.91
Heubach rotating drum		1.00	0.28	0.46
EN 15051 continuous drop			1.00	0.96
Palas single drop				1.00
